# The surface protein Shr of *Streptococcus pyogenes *binds heme and transfers it to the streptococcal heme-binding protein Shp

**DOI:** 10.1186/1471-2180-8-15

**Published:** 2008-01-23

**Authors:** Hui Zhu, Mengyao Liu, Benfang Lei

**Affiliations:** 1 Department of Veterinary Molecular Biology, Montana State University, Bozeman, Montana 59717, USA

## Abstract

**Background:**

The heme acquisition machinery in *Streptococcus pyogenes *is believed to consist of the surface proteins, Shr and Shp, and heme-specific ATP-binding cassette transporter HtsABC. Shp has been shown to rapidly transfer its heme to the lipoprotein component, HtsA, of HtsABC. The function of Shr and the heme source of Shp have not been established.

**Results:**

The objective of this study was to determine whether Shr binds heme and is a heme source of Shp. To achieve the objective, recombinant Shr protein was prepared. The purified Shr displays a spectrum typical of hemoproteins, indicating that Shr binds heme and acquires heme from *Escherichia coli *hemoproteins in vivo. Spectral analysis of Shr and Shp isolated from a mixture of Shr and heme-free Shp (apoShp) indicates that Shr and apoShp lost and gained heme, respectively; whereas Shr did not efficiently lose its heme in incubation with apoHtsA under the identical conditions. These results suggest that Shr directly transfers its heme to Shp. In addition, the rates of heme transfer from human hemoglobin to apoShp are close to those of simple ferric heme dissociation from hemoglobin, suggesting that methemoglobin does not directly transfer its heme to apoShp.

**Conclusion:**

We have demonstrated that recombinant Shr can acquire heme from *E. coli *hemoproteins in vivo and appears to directly transfer its heme to Shp and that Shp appears not to directly acquire heme from human methemoglobin. These results suggest the possibility that Shr is a source of heme for Shp and that the Shr-to-Shp heme transfer is a step of the heme acquisition process in *S. pyogenes*. Further characterization of the Shr/Shp/HtsA system would advance our understanding of the mechanism of heme acquisition in *S. pyogenes*.

## Background

Specific ATP-binding cassette (ABC) type transporters are involved in acquisition of essential iron in various forms in bacterial pathogens. The substrate-binding component of the ABC transporters specifically binds free Fe^3+^, ferric siderophore complex, or heme, which is transported across the cytoplasmic membrane by the permease component using the energy from the hydrolysis of ATP catalyzed by the ATPase component [1]. The human pathogen *Streptococcus pyogenes *produces three ABC transporters, FtsABCD [2], HtsABC or SiaABC [3,4], and MtsABC [5], which acquire ferric ferrichrome, heme, and Fe^3+ ^and Mn^2+^, respectively. Expression of *htsABC *and *mtsABC *are negatively regulated by the same metalloregulator MtsR in response to levels of Fe only and Fe or Mn, respectively [6,7].

Heme is abundant in mammalian hosts and a preferred iron source for bacterial pathogens [8-11]. Besides heme-specific ABC transporters, cell surface heme-binding proteins are required for heme acquisition in Gram-positive pathogens. These proteins have been identified in *S. pyogenes *[12], *Staphylococcus aureus *[13], and *Streptococcus equi *[14]. Heme is usually bound to host proteins at extremely high affinities [15,16]. Furthermore, Gram-positive bacteria have thick cell walls, and heme-specific ABC transporters are most likely buried in the cell wall. Thus, heme acquisition machinery in Gram-positive bacteria may have to have evolved mechanisms to overcome these obstacles in heme acquisition, i.e. inability of host hemoproteins to reach the ABC transporters and the extremely high affinity of host proteins for heme. The cell surface heme-binding proteins are believed to have evolved to overcome these obstacles in heme acquisition in Gram-positive pathogens. However, how these proteins are involved in heme acquisition is largely unknown.

*S. pyogenes *is capable of utilizing heme derived from human hemoproteins as a source of iron [9,17]. The heme acquisition machinery in *S. pyogenes *is believed to consist of Shr, Shp, and HtsABC. Shp and HtsABC are the cell surface heme-binding protein and heme-specific ABC transporter, respectively [4,12]. Shr, another cell surface protein, is proposed to be a receptor of host hemoproteins [3]; however, its function has not been firmly established. We have been studying the *S. pyogenes *heme acquisition machinery as a model system for understanding the heme acquisition process in Gram-positive pathogens. Shp rapidly and directly transfers its heme to HtsA, the lipoprotein component of HtsABC, in a concerted two-step process with one kinetic phase [18,19]. The structure of the Shp heme-binding domain reveals that the Shp heme iron is ligated to Met66 and Met153 [20], and the Met axial ligands are both important for rapid heme transfer from Shp to HtsA [21]. However, the heme source of Shp is not known.

We have proposed that Shp functions to relay heme from host proteins or another *S. pyogenes *heme-binding protein to HtsABC [18]. In this study, we found that the rates of ferric heme transfer from oxidized hemoglobin (methemoglobin) to heme-free Shp (apoShp) are similar to those for the dissociation of ferric heme from methemoglobin, suggesting that Shp cannot directly acquire heme from methemoglobin and that Shp may mainly acquire heme from another *S. pyogenes *protein. We hypothesize that this protein is Shr. To test this hypothesis, recombinant Shr was prepared and characterized. We found that Shr indeed binds heme and, more interestingly, efficiently transfers it to Shp but inefficiently to HtsA. These findings suggest the possibility that Shr is the heme source of Shp and that heme transfer from Shr to Shp is part of the heme acquisition process in *S. pyogenes*.

## Results

### Shp appears to indirectly acquire ferric heme from methemoglobin

**Figure 1:**
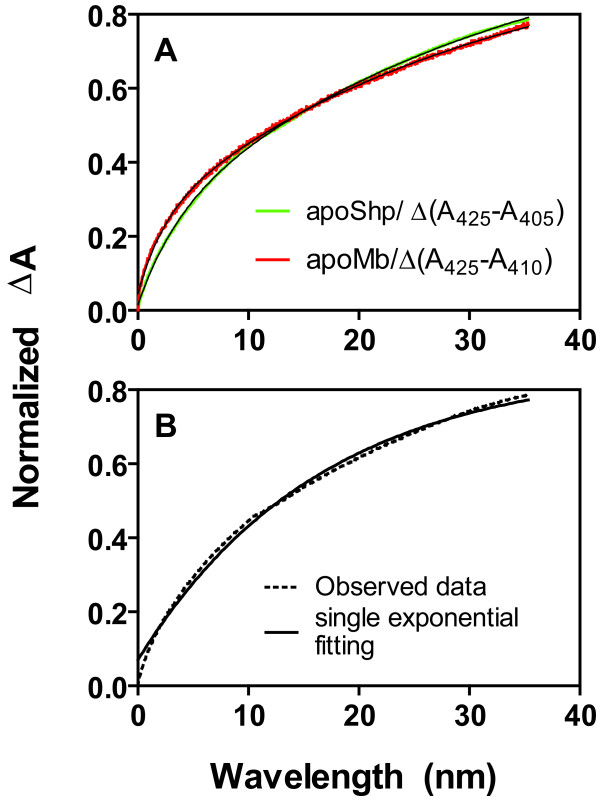
**Evidence for indirect heme transfer from human methemoglobin to apoShp**. Methemoglobin (total 5.8 μM heme) was reacted with 28 μM apoShp or 40 μM H64Y/V68F apomyoglobin in 20 mM Tris-HCl, pH 8.0, and 0.45 M sucrose, and absorbance time courses at 425 and 405 or 410 nm were recorded. (A) The normalized spectral changes Δ(A_425_-A_405_) (green curve) and Δ(A_425_-A_410_) (red curve) for the methemoglobin/apoShp and methemoglobin/apomyoglobin reactions, respectively. The black curves are the theoretical curves obtained by fitting the data to a two exponential equation. (B) Δ(A_425_-A_405_) of the methemoglobin/apoShp reaction does not fit a single exponential equation. The dashed and solid curves are respectively the observed data and theoretical curve from fitting of the data to a single exponential equation.

Heme-binding form of Shp (holoShp) was formed in the incubation of apoShp with human methemoglobin [18]. It was not known whether the formation of holoShp was due to direct heme transfer from methemoglobin or indirect scavenge of ferric heme dissociated from methemoglobin. To examine this issue, the rate for formation of holoShp was compared with that of heme dissociation from methemoglobin. Limited methemoglobin (total 5.8 μM heme) was incubated with 7 to 56 μM apoShp at 25°C, and the formation of holoShp was monitored by absorbance change at 405 and 425 nm, which present the loss and gain of heme by methemoglobin and apoShp, respectively. The time courses of normalized absorbance change, Δ(A_425_-A_405_), fit a two exponential equation (Fig. 1A), but not a single exponential equation (Fig. 1B). Fitting of the data to a two exponential equation resulted in two first-order rate constants. The values of the rate constants did not change significantly with [apoShp] from 7 to 56 μM, giving the mean values ± standard deviation of 0.0065 ± 0.0013 and 0.00041 ± 0.00012 s^-1 ^for the rate constants of the fast and slow phases, respectively. The biphasic kinetics and rate constants are similar to those in the dissociation of heme from methemoglobin using H64Y/V68F apomyoglobin as a heme scavenger [22], in which the fast and slow phases are heme dissociation from the β and α subunits, respectively [22,23]. To confirm these similarities, the dissociation of heme from methemoglobin using H64Y/V68F apomyoglobin was performed under the same conditions. Δ(A_425_-A_410_) associated with the loss and gain of heme by methemoglobin and apomyoglobin, respectively, was kinetically biphasic with 0.0058 ± 0.0002 and 0.00049 ± 0.00001 s^-1 ^for the rate constants of the fast and slow phases, respectively (Fig. 1A). These rate constants are close to those in the methemoglobin/apoShp reaction. These results suggest that Shp does not directly acquire heme from human methemoglobin.

### Recombinant Shr protein

**Figure 2:**
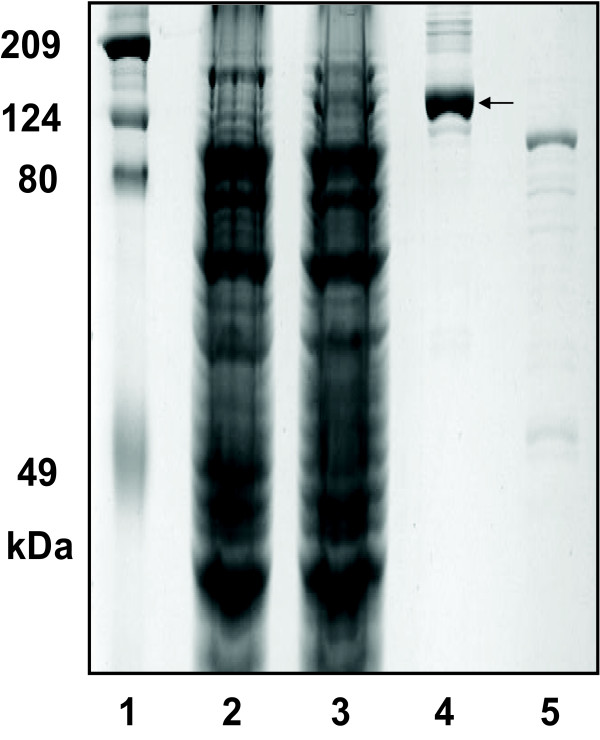
**SDS-PAGE showing expressed and purified recombinant Shr**. Lanes: 1, protein standards; 2, *E. coli *with empty vector (control); 3, *E. coli *containing Shr; 4, purified Shr; 5, proteins isolated from control *E. coli *using the first step of the Shr purification. The arrow indicates the Shr band.

**Figure 3:**
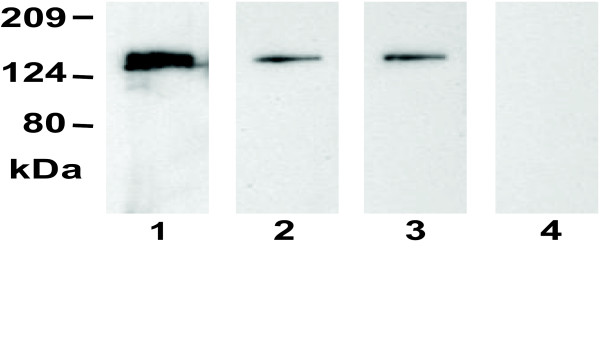
**Western blots showing the presence of Shr-specific antibodies in convalescent sera from patients of streptococcal pharyngitis**. Purified Shr (50 ng/lane) was resolved by SDS PAGE and analyzed by Western blotting using convalescent sera from three pharyngitis patients (lanes 1–3) and a person without *S. pyogenes *exposure as negative control (lane 4).

Since Shp appears not to directly acquire heme from methemoglobin, the heme source of Shp may be a *S. pyogenes *protein. We speculated that this protein was Shr. If this is true, Shr should bind heme and transfer it to Shp. To test this idea, we first prepared recombinant Shr protein. In comparison with *Escherichia coli *containing the empty vector, *E. coli *containing plasmid pET-His2-shr with the cloned *shr *gene displayed an extra band in SDS PAGE, which had approximately the expected molecular weight of Shr (theoretical molecular weight = 139,406) (Fig. 2). The band was obvious but not dominant, indicating moderate expression of Shr. The deduced amino acid sequence of the cloned *shr *gene has a 6xHis tag and predicted net charge of +7.6 at pH 7.0. As expected, the expressed protein bound to a cation exchange column of SP Sepharose at pH 8.0 and Ni^+^-NTA column. Shr obtained had a purity of 80% according to the densities of the bands in SDS-PAGE analysis (lane 4 in Fig. 2). To confirm that the protein purified was not an *E. coli *protein, the proteins of *E. coli*/pET-His2 (vector control) obtained using SP Sepharose chromatography, the first step in the Shr purification, was analyzed by SDS PAGE, and no protein with the size of Shr was detected (lane 5 in Fig. 2). The purified protein sample was also probed with convalescent sera from 3 patients of streptococcal pharyngitis and an individual without *S. pyogenes *exposure by Western blotting analysis. All three patients, but not the control individual, had antibodies specific for the purified protein (Fig. 3), further supporting that the purified protein was Shr. Thus, recombinant Shr can be expressed at moderate levels in *E. coli*.

### Shr binds heme

**Figure 4:**
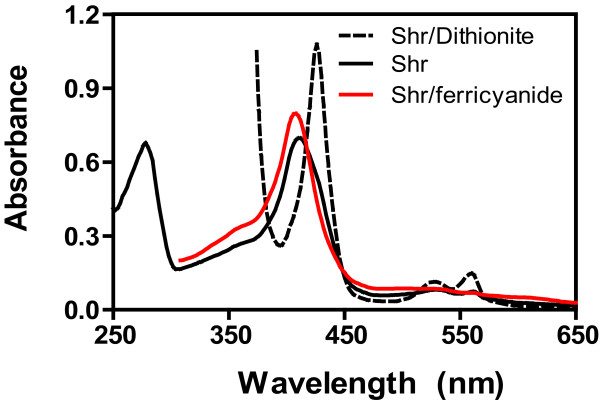
**Spectral evidence for that Shr binds heme**. Presented are the spectra of 15 μM Shr isolated (black solid curve), treated with 50 μM ferricyanide (red curve), and reduced by excess dithionite (dashed line) in 20 mM Tris-HCl, pH 8.0.

The pellet of *E. coli *expressing Shr was in light red color. Purified Shr was yellow and red at diluted and concentrated concentrations, respectively, while the proteins obtained from the control *E. coli *from the SP Sepharose chromatography did not have absorption in the visible region, indicating that Shr has an associated chromophore. In the presence of excess dithionite, the protein displays the absorption peaks at 426, 528, and 560 nm (Fig. 4), a spectrum typical of ferrous heme in a hexacoordinate form, indicating that the chromophore is heme. Shr as isolated had a broad Soret peak and the small resolved α band at 560 nm, suggesting that the protein was a mixture of ferric and ferrous heme complexes. To confirm this, the protein was incubated with ferricyanide, and excess ferricyanide was removed from the protein by a G-25 Sepharose column. The Soret peak of the ferricyaide-treated Shr was indeed shifted to that of ferric heme complex (Fig. 3), indicating that Shr as isolated was a mixture of ferric and ferrous heme complexes. The results of the pyridine hemochrome assay further confirm that the chromophore is heme. The spectrum of the chromophore derived from Shr in this assay was identical to the one of pyridine hemochrome derived from an authentic hemoprotein (data not shown). The molar ratio of associated heme to Shr was 0.8 after adjustment according to the estimated purity of the purified Shr. These results indicate that the chromophore associated with Shr is heme and that Shr can acquire heme from *E. coli *hemoproteins in vivo.

### Heme transfer from Shr to apoShp

**Figure 5:**
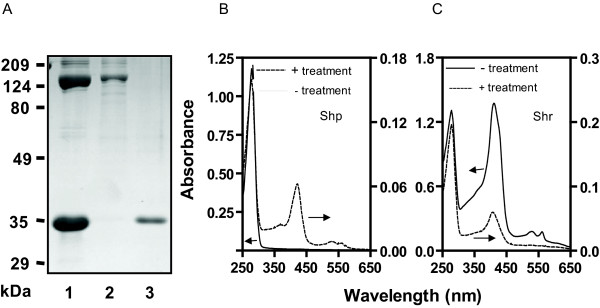
**Heme transfer from Shr to apoShp**. ApoShp(75 μM) was incubated with 10 μM Shr in 0.1 ml of 20 mM Tris-HCl, pH 8.0, at room temperature for 15 min. The sample was then loaded onto a 0.1-ml SP Sepharose column and eluted as described in the text. (A) SDS-PAGE analysis showing separation of Shr and Shp from their mixture. Lanes: 1, Shr/apoShp mixture before separation; 2, Shr isolated from the mixture; 3, Shp isolated from the mixture. (B) Spectra of apoShp (solid curve) and treated Shp (dashed curve). (C) Spectra of Shr (solid curve) and treated Shr (dashed curve). The arrows in panels B and C indicate the Y-axis of the corresponding spectrum.

We next tested whether Shr transfers its heme to apoShp. HoloShr (10 μM) and 75 μM apoShp were incubated for 15 min, and the two proteins were separated as described in the Methods section. SDS-PAGE analysis confirmed the separation of the two proteins (Fig. 5A). HoloShp was present in the isolated Shp sample, as evidenced by the presence of absorption of bound heme (Fig. 5B). Thus, apoShp gained heme in its reaction with Shr. The ratio of A_410_/A_280 _of the treated Shr was 29% of that of the starting Shr (Fig. 5C). A_410 _is the absorbance of bound heme, and A_280 _is mainly the absorbance of the protein moiety. Thus, A_410_/A_280 _can be used to approximately estimate relative levels of heme in the protein samples. The lower A_410_/A_280 _ratio of the treated Shr indicates that Shr lost heme in its reaction with apoShp. These results suggest that Shr transfers its heme to apoShp.

### Shr does not efficiently transfer its heme to apoHtsA

**Figure 6:**
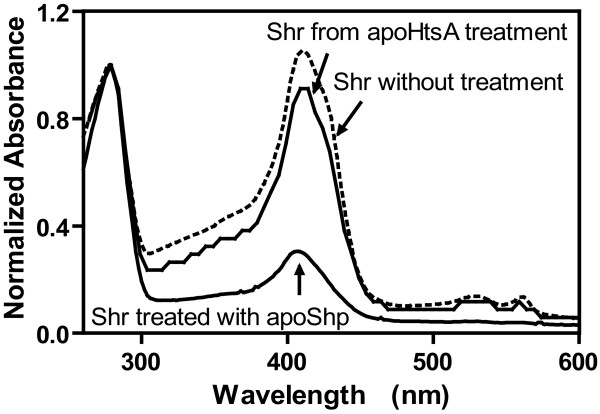
**Inefficient heme transfer from Shr to apoHtsA**. ApoHtsA or apoShp (75 μM) was incubated with 15 μM Shr in 0.1 ml of 20 mM Tris-HCl, pH 8.0, at room temperature for 2 min. Each sample was then loaded onto a 0.1-ml SP Sepharose column and eluted as described in the text. Presented are the normalized spectra of Shr without or with treatment with apoHtsA or apoShp. The normalization was done by setting A_280 _= 1.0.

To test whether Shr transfers its heme to apoHtsA, 15 μM holoShr was incubated with 75 μM apoHtsA or apoShp (positive control) for 2 min, and Shr was isolated from the mixtures. The normalized spectrum of the isolated Shr from its apoHtsA mixture was compared with those of untreated Shr and Shr isolated from its mixture with apoShp. As shown in Fig. 6, the ratio of A_410_/A_280 _of Shr from the Shr/apoHtsA reaction was decreased from 1.05 to 0.91, whereas the A_410_/A_280 _ratio of Shr from the Shr/apoShp reaction was lowered from 1.05 to 0.3. According the extinction coefficients of holoShr and apoShr at 280 and 408 nm and these A_410_/A_280 _ratios, Shr lost 14% and 73% heme in its reactions with apoHtsA and apoShp, respectively. These results indicate that Shr transfers its heme to apoShp more efficiently than to apoHtsA.

## Discussion

One of the major findings of this study is that Shr binds heme. Purified Shr has a chromophore and displays a visible absorption spectrum typical of hemoproteins. Reduction of Shr with dithionite results in the spectrum typical of bound ferrous heme in a hexacoordinate complex. The hemochrome assay also resulted in hemochrome from Shr. These findings indicate that Shr binds heme and can acquire heme from *E. coli *hemoproteins.

Andrade *et al*. [24] identified a domain in genes near the components of a putative Fe^3+ ^siderophore transporter (designated NEAT domain) from Gram-positive pathogenic bacteria. It should be pointed out that the transporters in *S. pyogenes *and *S. aureus *referred in the work of Andrade et al. were later found to be heme transporters [4,13]. Shr, which was referred to S_pyog in this reference, has two NEAT domains. The NEAT domain in *S. aureus *surface proteins IsdA and IsdC binds heme [25,26], whereas the NEAT domain of IdsH does not bind heme [27], suggesting that the binding of heme to Shr may be mediated by one or both NEAT domains.

Another major finding is that Shr appears to directly transfer its heme to apoShp. When Shr was incubated with apoShp or apoHtsA under the same conditions, the majority of Shr lost heme to apoShp, but not to apoHtsA. The apparent bimolecular rate constant for binding of ferric heme to apoHtsA at low [ferric heme] (*k *= 80 μM^-1^s^-1^) is 50 times as that for binding of ferric heme to apoShp (*k *= 1.6 μM^-1^s^-1^), and the association constant for HtsA-ferric heme complex (K = 31,000 μM^-1^) is 5-fold greater than that for Shp-ferric heme complex (K = 5,300 μM^-1^) [19]. Based on these previous kinetic and equilibrium constants, the efficient and inefficient heme transfer from Shr to apoShp and from Shr to apoHtsA, respectively, suggests that Shr directly transfers its heme to apoShp and indirectly and inefficiently to apoHtsA. Otherwise, Shr would release heme into solvent, which should be caught more efficiently by apoHtsA than apoShp, and Shr should thus lose heme to apoHtsA and apoShp at least to similar extent.

The dissociation of ferric heme from methemoglobin has been found to be a slow process with two kinetic phases using H64Y/V68F apomyoglobin as a heme scavenger [22]. In this reaction, methemoglobin dissociates heme into solvent, which is caught by the mutant apomyoglobin, and the fast and slow phases are the dissociation of heme from the β and α subunits, respectively [22,23]. The time courses of absorbance change in the reactions of methemoglobin with apoShp and apomyoglobin were almost overlaid with similar rate constants. In addition, the rate constants of the two kinetic phases of the methemoglobin/apoShp reaction did not significantly change with [apoShp]. These observations suggest that apoShp does not directly, but indirectly, get heme from methemoglobin. Thus, the heme source of Shp in vivo is most likely Shr.

We have reported the direct heme transfer from Shp to HtsA [18,19], the only example of heme transfer from a surface protein to ABC transporter. Now we have found heme transfer from a cell surface protein to another cell surface protein. These new findings provide opportunity to fully elucidate the molecular mechanism of heme acquisition in *S. pyogenes*. We propose that Shr acquires heme from host proteins and that Shp relays heme from Shr to HtsA. We are currently in process to investigate whether methemoglobin directly transfers its heme to Shr and whether Shp facilitates heme transfer from Shr to HtsA. Although *S. pyogenes *Shr and Shp have homolog only in *S. equi *[14] among the bacteria with known genome sequences currently, the hemoglobin receptor IsdB of *S. aureus *[28] is a cell surface protein and binds heme [13]. *S. aureus *has additional cell surface heme-binding proteins. It is most likely that *S. pyogenes *and *S. aureus *follow similar mechanism of heme transfer, although the proteins involved are not homologous in amino acid sequence. This idea is supported by the similarity in the structures of the Shp heme-binding domain and the NEAT domain of IsdA and IsdC [20,25,26] and by the homology between *S. pyogenes *HtsA and *S. aureus *IsdE [29]. Indeed, IsdA rapidly and directly transfers its heme to IsdC in a kinetic mechanism [30] that is similar to that of heme transfer from Shp to HtsA [19]. Thus, the detailed biochemical mechanism of heme transfer in the *S. pyogenes *heme acquisition system may serve as a model for heme acquisition in Gram-positive pathogens.

## Conclusion

Shr binds heme in a hexacoordinate form and acquires heme from *E. coli *hemoproteins in vivo. Shr efficiently transfers its heme to Shp, but not to HtsA, under the same conditions, suggesting that Shr-to-Shp heme transfer is direct. In contrast, Shr does not directly acquire heme from human methemoglobin. These results suggest the possibility that Shr, but not methemoglobin, is a source of heme for Shp in vivo and that the Shr-to-Shp heme transfer is a step of the heme acquisition process in *S. pyogenes*.

## Methods

### Materials

All chemicals were purchased from Sigma (St. Louis, Mo.). Pfu Ultra high-fidelity DNA polymerases used for PCR cloning was from Stratagene. SP Sepharose, DEAE Sepharose, and phenyl Sepharose were obtained from GE Healthcare (Piscataway, NJ). *E. coli *strains NovaBlue and BL21 (DE3) (Novagen, Madison, Wis.) were used as hosts for gene cloning and protein expression, respectively. All solutions used in protein purification and heme-transfer reactions were buffered with 20 mM Tris-HCl, pH 8.0. Human hemoglobin was purchased from ICN Pharmaceuticals, Inc. Heme-free H64Y/V68F sperm whale myoglobin was prepared as described [22].

### Gene cloning

The *shr *gene was cloned from a serotype M1 GAS strain MGAS5005 [31] in two steps because of its large size. The 3' fragment of *shr *was amplified by PCR with primers 5'-CAGCCATGGGAGGTCAAGAAAGTG-3' and 5'-TGAATTCTAGTGATAACGAGAGCTACTTTC-3'. The PCR product was digested with *Nco*I and *Eco*RI and ligated into pET-His2 [31] to create pET-His2-shr_3'*f*_. The 5' fragment of *shr *was amplified using primers 5'-ATCCATGGCTCAAACTGTAAAATCACAAGAG-3' and 5'-CTCCCATGGCTGATACCAAAAC-3' and cloned into the *Nco*I site of pET-His2-shr_3'*f*_, to yield pET-His2-shr. No amino acid replacement was introduced by this strategy. The cloned *shr *gene had no spurious mutations according to DNA sequencing. The proteins made from pET-His2-shr lacked the putative secretion signal sequence (amino acids 1–21) and the charged tail and transmembrane domain at the carboxyl terminus (amino acids 1252 to 1275) and had 12 amino acid residues, MHHHHHHLETMA, fused to the first amino acid residue (Q22) of the mature protein.

### Expression and purification of recombinant Shr

For expression of Shr, *E. coli *BL21 (DE3) cells containing pET-His2-shr were grown in 8 liters of Luria-Bertani broth supplemented with 100 mg/l ampicillin at 37°C to an OD_600 _of approximately 1.0, and Shr expression was induced with 0.5 mM IPTG for 8 h. Bacteria were harvested by centrifugation for 10 min at 4,000 × *g*. The cell paste was resuspended in 120 ml of Tris-HCl containing 1 mM phenylmethylsulfonyl fluoride, a protease inhibitor, sonicated on ice for 15 min, and centrifuged at 15,000 × *g *for 20 min to remove cell debris. The supernatant was loaded onto a SP Sepharose column (2 × 10 cm) equilibrated in Tris-HCl and washed with 100 ml Tris-HCl. The protein was eluted from the column with a 200-ml gradient of 0 to 500 mM NaCl. The fractions containing Shr were pooled, dialyzed against Tris-HCl, loaded onto a DEAE Sepharose column (1.5 × 4 cm), and washed with 100 ml Tris-HCl. Flow-through containing Shr was collected. (NH_4_)_2_SO_4 _was added to the flow-through to a final concentration of 1.2 M, the sample was applied onto a phenyl Sepharose column (1.5 × 10 cm), and the column was washed with 50 ml of 1.0 M (NH_4_)_2_SO_4 _and eluted with a 70-ml linear gradient of 1.0 M to 0.2 M (NH_4_)_2_SO_4_. Fractions containing Shr were pooled and dialyzed against 3 l Tris-HCl at 4°C for 24 h. Shr was concentrated using Centricon Plus 20 filtration devices (Millipore, Bedford, MA).

### Preparation of apoShp

Recombinant Shp was expressed as described previously [12]. Portion of recombinant Shp was in inclusion bodies and recovered as pellet after lysis by sonication. Insoluble Shp from 6 liters of culture was dissolved in 20 ml of 8 M urea, and the denatured Shp was refolded by dialysis against 3 l Tris-HCl. The refolded Shp was loaded onto an SP Sepharose column (2.5 × 3 cm), and the column was washed with 50 ml Tris-HCl and eluted with a 100-ml linear gradient of 0 to 150 mM NaCl. Fractions containing apoShp were pooled, dialyzed against 3 l Tris-HCl, and loaded onto a DEAE Sepharose column (2.5 × 2.5 cm). The column was washed with 50 ml Tris-HCl and eluted with a 100-ml linear gradient of 0 to 300 mM NaCl. Shp eluted was dialyzed against Tris-HCl and concentrated using Centricon Plus 20 filtration devices. Shp obtained was in apo-form and had a purity of >95% according to SDS-PAGE analysis.

### Western blotting analysis

The presence of Shr-specific antibody in convalescent sera of patients was assessed by Western blotting analysis, as described previously [32]. Convalescent-phase serum samples were obtained from 3 patients with streptococcal pharyngitis at about 21 days after diagnosis. The protocol for the collection and analysis of the sera was approved by the Institutional Review Board at Montana State University, Bozeman.

### Determination of heme and protein contents

Protein concentrations were determined using a modified Lowry protein assay kit (Pierce, Rockford, IL) with bovine serum albumin as a standard. Heme content was measured using a pyridine hemochrome assay [33]. Briefly, protein sample in 750 μl of Tris-HCl was mixed with 175 μl pyridine, 75 μl of 1 N NaOH, and approximately 2 mg sodium hydrosulfite. The optical spectrum was then immediately recorded. The heme content was determined using the extinction coefficient ε_418 _= 191.5 mM^-1 ^cm^-1^. All absorption spectra were obtained using a SPECTRAmax 384 Plus spectrophotometer (Molecular Devices, Sunnyvale, CA).

### Heme transfer from human methemoglobin to apoShp

The procedure for measurement of heme dissociation from methemoglobin using H64Y/V68F apomyoglobin as a heme scavenger [22] was followed for the hemoglobin/apoShp reaction. Methemoglobin (total 5.8 μM heme) was reacted at 25°C with 7, 14, 28, or 56 μM apoShp in 20 mM Tris-HCl and 0.45 M sucrose. Absorbance traces at 405 and 425 nm were recorded. Spectral change Δ(A_425_-A_405_) was analyzed by fitting to a two exponential expression to get two rate constants for the fast and slow phases. As a control, the reaction of methemoglobin (5.8 μM heme) with 40 μM H64Y/V68F apomyoglobin was also performed under the identical conditions, and absorbance traces at 410 and 425 nm were recorded and analyzed as in the methemoglobin/apoShp reaction.

### Heme transfer from Shr to apoShp or apoHtsA

Shr at 10 or 15 μM was incubated with 75 μM apoShp or apoHtsA at 25°C for 2 or 15 min. The proteins in the mixtures were separated as follows. In the Shr/apoShp reaction, the incubated mixture was loaded onto a small SP Sepharose column (~0.1 ml resin), and the column was washed with 2 ml Tris-HCl. Shp was first eluted from the column with 2 ml of 50 mM NaCl, and Shr was then eluted with 2 ml of 200 mM NaCl. In the Shr/apoHtsA reaction, the mixture was loaded onto a small SP Sepharose column (~0.1 ml resin), and the column was washed with 2 ml Tris-HCl. HtsA did not bind to the column and was washed out by Tris-HCl, and Shr was then eluted out with 2 ml of 200 mM NaCl. The separation of the two proteins was confirmed by SDS-PAGE analysis, and the loss and gain of heme by Shr and apoShp or apoHtsA, respectively, were assessed by UV-visible spectra of the separated proteins.

### Nucleotide sequence accession number

The GenBank accession number for the nucleotide sequence of the *S. pyogenes shr *gene is EU098089.

## Authors' contributions

HZ cloned the *shr *gene, performed the Shr-to-apoShp heme transfer, and participated in drafting the manuscript. ML purified recombinant Shr and conducted the Shr-to-apoHtsA heme transfer. BL designed the study, performed the methemoglobin/apoShp and methemoglobin/apomyoglobin reactions, and drafted the manuscript. All authors have read and approved the final manuscript.
